# Malformation lymphatique macrokystique atypique chez un adulte

**DOI:** 10.11604/pamj.2017.28.128.13472

**Published:** 2017-10-10

**Authors:** AHind Ramid, Fouzia Hali

**Affiliations:** 1 Service de Dermatologie-Vénéréologie, CHU Ibn Rochd, Université Hassan II, Casablanca, Maroc

**Keywords:** Malformation lymphatique, lymphangiome, lymphangiome macrokystique, Lymphatic malformation, lymphangioma, macrocystic lymphangioma

## Abstract

Macrocystic lymphatic malformations (MLMs) constitute a circumscribed variation in deep lymphangiomas. They are characterized by a rare occurrence in adults, by their rapid expansion and by their frequent occurrence at the level of the cervicofacial region (75%) and axillary region (20%). They rarely occur in lower limbs. The diagnosis of MLMs is primarily based on clinical examination. Ultrasound has a valuable role in positive diagnosis and sometimes in differential diagnosis. MRI has a role in the diagnosis and the assessment of tumor extent, thus making a valuable contribution to surgery. Therapeutically, MLMs are mainly treated by sclerotherapy; surgery is rarely indicated. We report the case of 26-year old man with a one-year history of huge painless and rapidly evolving subcutaneous swelling in the right thigh. Clinical examination showed circumferential compressible non-beating infiltrated mass, surmounted by vegetative and ulcerated lesions. The remainder of the clinical examination showed the presence of inguinal ipsilateral lymphadenopathy, with mobility limitation of the right knee. Angio-MRI objectified diffuse heterogeneous gadolinium-enhancing cutaneous, subcutaneous thickening of the right thigh with perimuscolar aponeurotic involvement. Lymphoscintigraphy showed asymmetry of the accumultion of radiopharmaceutical kinetics that was reduced in the right lower limb in favor of an overload of the lymphatic system at rest. Given the clinico-radiological data, the diagnosis of MLM was retained. Given the seat of the lymphangioma and its extent, after consultation with the surgeons there was no indication for surgery. Immobilization with a removable bandage was indicated for our patient.

## Image en médecine

Les malformations lymphatiques macrokystiques (MLMK) constituent une variante circonscrite des lymphangiomes profonds. Elles se caractérisent par leurs survenue rare chez l’adulte, leurs expansion rapide et leurs fréquence au niveau de la région cervicofaciale (75%) et axillaire (20%), l’atteinte du membre inférieur est exceptionnelle. Le diagnostic des MLMK est essentiellement clinique, l’échographie a un intérêt pour le diagnostic positif et parfois différentiel. L’IRM a une place dans le diagnostic et l’appréciation des limites de la tumeur apportant ainsi une aide précieuse à la chirurgie. Sur le plan thérapeutique, les MLMK relèvent plutôt d’une sclérothérapie, rarement la chirurgie est indiquée. Nous rapportons le cas d’un homme de 26 ans qui présentait depuis un an une énorme tuméfaction sous-cutanée de la cuisse droite, indolore et rapidement évolutif. L’examen clinique trouvait une masse circonférentielle infiltrée, compressible et non battante, surmontée de lésions végétantes et ulcérées. Le reste de l’examen clinique notait la présence d’adénopathie inguinale homolatérale avec limitation de la mobilité du genou droit. L’angio IRM objectivait un épaississement cutanéo-sous cutané diffus de la cuisse droite d’aspect hétérogène et prenant le contraste avec atteinte aponévrotique périmusculaire. La lymphoscintigraphie révélait une asymétrie de la cinétique d’accumulation du radiopharmaceutique au détriment du membre inférieur droit en faveur d’une surcharge du système lymphatique au repos. Devant les données clinico-radiologiques, le diagnostic de MLMK a été retenu. Après concertation avec les chirurgiens il n’y avait pas d’indication chirurgicale vue le siège du lymphangiome et son étendu; une contention par des bandes amovibles était indiquée chez notre patient.

**Figure 1:**
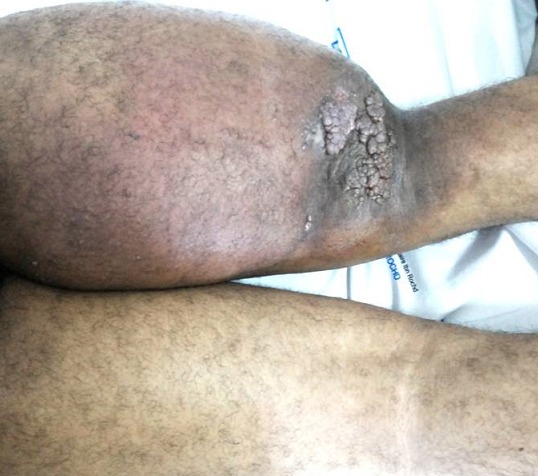
Lymphangiectasies au niveau de la face postéro-inférieure de la cuisse

